# Brain responses to social punishment: a meta-analysis

**DOI:** 10.1038/s41598-019-49239-1

**Published:** 2019-09-05

**Authors:** Oksana Zinchenko

**Affiliations:** Centre for Cognition and Decision Making, Institute for Cognitive Neuroscience, National Research University Higher School of Economics, Moscow, Russian Federation

**Keywords:** Decision, Cooperation

## Abstract

Many studies suggest that social punishment is beneficial for cooperation and consequently maintaining the social norms in society. Neuroimaging and brain stimulation studies show that the brain regions which respond to violations of social norms, the understanding of the mind of others and the executive functions, are involved during social punishment. Despite the rising number of studies on social punishment, the concordant map of activations - the set of key regions responsible for the general brain response to social punishment - is still unknown. By using coordinate-based fMRI meta-analysis, the present study examined the concordant map of neural activations associated with various social punishment tasks. A total of 17 articles with 18 contrasts including 383 participants, equalling 191 foci were included in activation likelihood estimation (ALE) analysis. The majority of the studies (61%) employed the widely used neuroeconomic paradigms, such as fairness-related norm tasks (Ultimatum Game, third-party punishment game), while the remaining tasks reported criminal scenarios evaluation and social rejection tasks. The analysis revealed concordant activation in the bilateral claustrum, right interior frontal and left superior frontal gyri. This study provides an integrative view on brain responses to social punishment.

## Introduction

Cooperation is one of the mechanisms supporting the social order in society. The emergence and enforcement of cooperation in groups is one of the most fundamental questions, and many studies shed light on factors which are important to sustain and enforce cooperation in society, such as reciprocity and social punishment as a form of negative reciprocity^1,2^. Social punishment is a sanctioning behaviour that occurs when a person with no apparent benefit (or even at a cost) to himself, punishes deviant behaviour that violates existing social norms^3^. It exists in different forms, such as second-party and third-party punishment which is usually implemented in economic tasks, or as acts of aborting social interaction - social disapproval or rejection. When the punishment decision is implemented by the person who is affected during the norm violation, it is called “second-party punishment”, otherwise if the person is uninvolved, but knows about the norm violation – it is called “third-party punishment”^4^. Behavioural economists suggest that third-party punishment may have emerged from second-party punishment^5,6^. Thus, we could suggest that it would have some shared neural representations.

Recent studies suggest that two main forms of social punishment – second-party and third-party punishment – share some common brain mechanisms, such as involvement of ventral striatum^7,8^. However, the studies also report differences in the neural representation for these two forms of punishment: medial prefrontal cortex^8,9^, right nucleus accumbens and bilateral cingulate^7^, right dorsolateral prefrontal cortex (rDLPFC), left anterior insula and amygdala^8^. While looking for behavioural implications, Stallen *et al*.^8^ showed, using a computational model, that willingness to punish in response to unfairness did not differ between second- and third-party punishment games; however, the severity of punishment is significantly higher for second-party situations. At the neural level the willingness to punish in both second- and third-party situations was associated with the right anterior insula’s activation^8^, while the activation of left anterior insula, rDLPFC and left amygdala and was specific for second-party’s willingness to punish. Based on these findings, we expect that tasks related to social punishment will show concordant brain locations in ventral striatum and right anterior insula.

In addition, a recent review by Krueger and Hoffman^6^ provides us with a neural framework for punishment that could offer some insights regarding the involvement of large-scale networks supporting third-party punishment: the salience network anchored in the anterior cingulate cortex, the mentalizing network – in the temporoparietal junction and dorsomedial prefrontal cortex and central-executive network – in the dorsolateral prefrontal cortex. Recent fMRI study by Civai and colleagues^10^ suggests that exploration of the similarities in neural processing of second-party and third-party punishment conditions could deep our knowledge in disentangling mechanisms associated with general punishment processes. However, no extensive review or meta-analytic study has been done on social punishment to address shared brain mechanisms for both second- and third-party punishment: a recent ALE meta-analysis was conducted on social norm representation and norm violations^11^, revealing the distinct brain regions responsible for these two processes – the anterior cingulate cortex, medial frontal gyrus and insular cortex. The majority of the experiments testing social punishment focus on punishment in terms of direct material costs alone. However, initially^12^ this has been done to control individual incentives and test exact theoretical predictions, while laboratory behaviour of meeting and avoiding punishment is similar to the disapproval and its avoidance in real life. Thus, social punishment should be seen more as different forms of social control^13^, or judgments of disapproval and negative emotional responses to the norm violator^14^, so the meta-analytic research should follow this logic to better estimate concordant activation to social punishment. Following this, the search for the current study was constructed to address different terms used to describe social punishment: social punishment, altruistic punishment, costly punishment - so different tasks from the punishment experiments would be included in the study to replicate a mechanism that supports cooperation and enforces social norms. The initial literature search performed in April, 2017, did not reveal enough studies eligible to be included in the analysis of general brain responses to social punishment. This study is an attempt to address this lack of knowledge and identify the concordant brain activations responsible for the processing of information related to social punishment.

## Results

**Figure 1:**
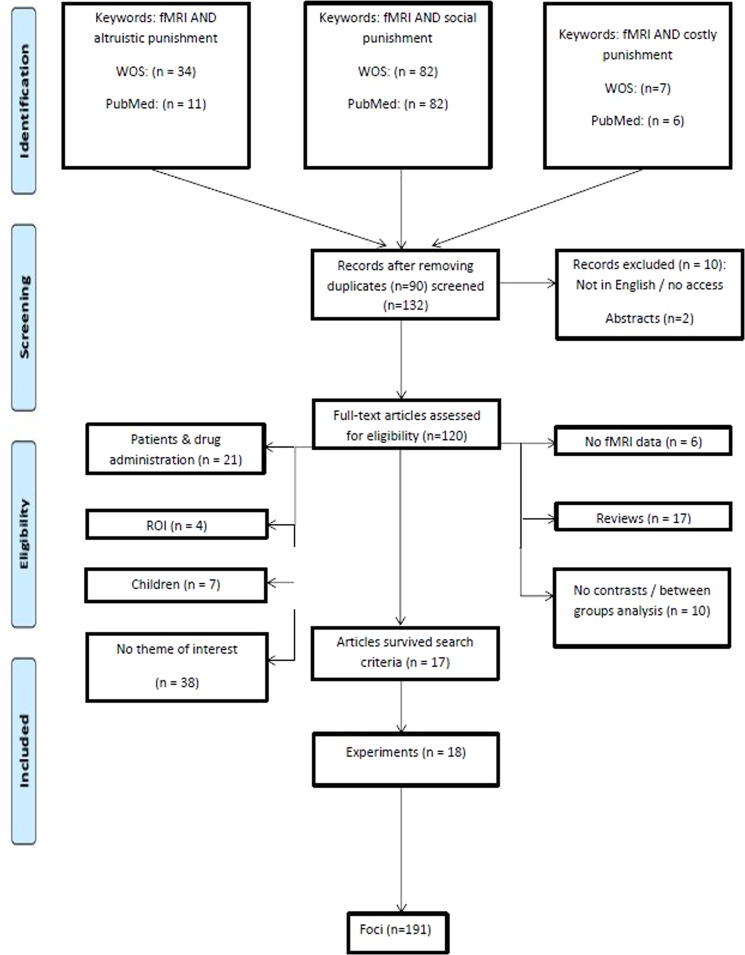
The PRISMA flowchart for the search and eligibility of the articles^69^.

Articles included in the meta-analysis provided data on 383 participants (see Table 1). Five articles did not report the gender of their final sample after all exclusions; of the remaining articles, 57% were female participants. Four articles did not report handedness (25%); of the remaining articles all tested participants were right-handed (100%). Five articles did not report the age of the final sample of the participants; of the remaining articles, the mean age range was 19.76–28.39 years. 50% of the articles did not report the education level of participants, while 100% of the participants of the remaining articles were reported to have some university education. Figure 1 shows the number of articles, number of studies (experiments) and number of foci included in the meta-analysis.

**Table 1:** Descriptive information of studies and experiments included.

Author, year	N	F	Education	Handedness	Age	Task	Contrast	Foci
Baumgartner, 2012	16	0	n/r	right	24.5 ± 2.2	Third-party punishment task	Outgroup(BC) > Ingroup(AC + ABC)weighted	8
Bellucci, 2016	26	13	university	right	26.0 ± 5.7	Criminal scenarios (vignettes) evaluation and punishment assignment as a third-party	Experimental > Control	6
Buckholtz, 2008	16	8	n/r	right	18–42	Third-party legal decision-making task (scenarios evaluation)	High > low punishment	11
Corradi-dell’Acqua, 2013	23	9	n/r	n/r	18–35	Ultimatum Game	Rejected > accepted unfair offers	2
Feng, 2016	22	11	university	right	22.9 ± 1.6	Third-party punishment task	Unfair > fair	19
Guo, 2013	21	n/r	university	right	n/r	Ultimatum Game	(Reject-Accept)Unfair Loss > (Reject-Accept)Unfair Gain	20
Hu, 2015	25	n/r	n/r	n/r	n/r	Third-party help and punishment task	Punish > punish control	8
Kohls, 2013	22	11	university	n/r	25.6 ± 3.5	Social incentive delay task (SID)	Anticipation avoi > con	6
							Outcome avoi > con	2
Moor, 2012	15	8	n/r	right	20.38 ± 0.85	Cyberball paradigm & punishment questionnaires	Team2 > team1 (19–21 years old)	5
Spitzer, 2007	23	n/r	university	right	n/r	Monetary task with control and punishment conditions	Punishment > Control	20
Strobel, 2011	24	11	university	n/r	23.8 ± 3.8	First-person and third-person Dictator Game with punishment option	Punishment > no punishment	17
Treadway, 2014	30	10	n/r	right	18–30	Scenarios evaluation and punishment assignment	Punished > Not punished trials	15
Vrticka, 2008	16	8	n/r	right	23.6 ± 3.6	Perceptual task with social feedback	Angry > Smiling Faces with Lost Feedback	3
Wang, 2017	26	15	university	right	20.92 ± 2.04	Third-party punishment task	Unfair > fair	9
Wei, 2018	25	n/r	university	right	n/r	Modified Ultimatum Game	Unfair > fair	6
Will, 2015	26	16	n/r	right	20.7 ± 1.97	Cyberball & Dictator Game	Percentage inequality for excluders > percentage inequality for includers *	33
Wu, 2015	27	n/r	university	right	n/r	Ultimatum Game & Dictator Game	DG following the unfair UG > DF following the fair UG	1

Note: n/r: not reported. Age is reported either in mean and standard deviation or in range. * derived by whole-brain contrast, positive correlation with punishment frequency.

## ALE map

**Figure 2:**
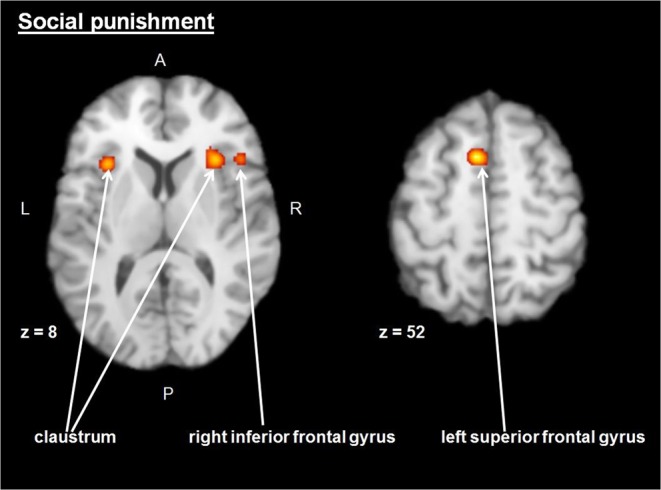
Brain map of significant ALE values for social punishment category. Left = left. Coordinates are presented in Talairach space.

Two clusters were detected during the analysis (see Fig. 2, Table 2; the coordinates are in Talairach space). One with the highest likelihood of detection includes right claustrum and right inferior frontal gyrus (BA 45). Other regions include the left claustrum and left superior frontal gyrus (BA 6). The figure was prepared using Mango software v. 4.0.1. (freely available at http://ric.uthscsa.edu/mango/).

**Table 2:** Concordant areas for “social punishment” category.

Category	Volume mm^3^	ALE Value	x	y	z	Brain Area	BA
Social punishment	2208	0.022	30	18	8	Right Claustrum	
		0.020	46	20	10	Right Inferior Frontal Gyrus	45
	1376	0.024	−32	16	4	Left Claustrum	
	1256	0.026	−6	10	52	Left Superior Frontal Gyrus	6

## Discussion

The analysis revealed concordant bilateral activation in the claustrum, left superior frontal gyrus and right inferior frontal gyrus for social punishment tasks. The former corresponds to the region next to the insular cortex, which is considered in Krueger and Hoffman’s model as part of the salience network. The latter corresponds to the dorsolateral and ventrolateral prefrontal cortex, respectively, while the dorsolateral prefrontal cortex represents the main node of central-executive network in Krueger and Hoffman’s model. It needs to be taken into account that the analysis did not reveal concordant activation in any regions corresponding with the mentalizing network from the model. TPJ is a key node of the mentalizing network included in the model, whose activity is associated with the evaluation of the wrongdoer’s mental state and his/her intention^6,15^. This could be explained by the heterogeneity of the studies included: the proportion of third-party and second-party punishment tasks is not equal, and it has been shown that second-party and third-party punishment tasks trigger different responses in the mentalizing system^9,16^. The analysis did not reveal any concordant activation in ACC responsible for the initial evaluation of harm to the victim of the norm violation, according to the Krueger and Hoffman model. In general ACC activity is associated with error detection and performance monitoring^17–19^. For social punishment, this suggests that ACC activity is required for the initial stages of punishment decision-making, like the evaluation of the current norm and a detection of the norm violation, which is consistent also with the results of the recent meta-analysis^11^. As most studies included in the current meta-analysis used the contrasts where the participants were faced with the punishment decision of others/make the punishment decision by themselves (assigning the rating, deducting the monetary units etc.), this could suggest that the final stages of punishment decision-making only require already available information about the harm done to the victim (associated with the insular/claustrum activity) to make the final decision (which involves lateral prefrontal cortex/inferior frontal gyrus activity).

The right inferior frontal gyrus is considered as a key region in the emotional empathy network^20–23^. The activation of this region has been also associated with interpretation during interpersonal interactions^24^ and an understanding of the intentions of others^25^. The structural abnormalities of this region have been reported in association with the severity of symptoms in autism spectrum disorders, such as reduced cortical thickness^26^ or the absence of functional activation^27^. Based on these findings, it could be suggested that the activation of this part of the ventrolateral prefrontal cortex reflects the activation of the emotional empathy network required for the adequate response to social interaction.

According to a recent meta-analysis, the concordant activation of the left claustrum is associated with norm violation tasks^11^, although its direct role in social cognition is still unclear. However past hypotheses have suggested the role of the claustrum in mammals as an integrator of multimodal sensory information, though some recent findings do not confirm this notion^28,29^; this suggests its involvement in top-down control and encoding a preparatory signal in rat models^28^.

It is important to note that emotional empathy networks, based on the concept of mirror neuron systems, are now claimed to include regions of the somatosensory cortex and anterior insula^30^. Recent findings suggest that the right inferior frontal gyrus response triggers the activity in the anterior insula associated with emotional simulation^20^, confirming its interaction during the evaluation of social-emotional events. Moreover, some researchers claim that the role of the anterior insula as a hub of salience networks could be confounded by its proximity to the claustrum^31^, which is supported by findings on monkeys^32,33^, suggesting that claustrum reacts to the novel and salient stimuli. Based on this, it could be suggested that activations of the claustrum in social punishment tasks are a function of salience detection.

The analysis also revealed the concordant activation in left superior frontal gyrus (BA 6) for social punishment. This area could be divided into three functional subdivisions - supplementary motor area, the dorsal and ventral parts of lateral premotor cortex^34^. The findings suggest that various tasks could activate different parts of this area. The medial part of BA 6 corresponds to the supplementary motor area, whose main functions are motor learning and planning^35^, however, as part of DLPFC, is it also involved in working memory^36,37^. Lesion studies suggest that the impairment of the left superior frontal gyrus is associated with working memory deficits^38^. Schizophrenia patient studies also confirm the role of this region in working memory, showing a higher activation of the left superior frontal gyrus during more complex working memory tasks^39^. The involvement of the activity of this region in working memory tasks is also supported by findings on healthy subjects, addressed in particular to task-switching^40^. The rostral part of BA 6, however, is activated in mental-operation tasks without motor responses involved^34^. Some studies also linked BA 6’s activation with number multiplication and comparison^41^ and spatial imagery^42^, which suggests that the activation of this area could be responsible for the interaction of cognitive information processing and motor control.

The data presented here represent concordant neural activation across studies that mostly investigated the brain responses of punishers (fifteen studies out of seventeen) using neuroeconomic paradigms (eleven out of seventeen). The optimal approach would be possible with the further implementation of subcategories (i.e. second- vs third-party punishment; experience of receiving the punishment vs application of the punishment to the other person). In accordance with current guidelines^43^, the number of experiments in these subcategories does not allow us to continue the examination of the concordance.

The current study employed ALE approach to investigate concordant activations to social punishment, following the model of Krueger and Hoffman. An alternative solution would be to perform additional analysis, such as Meta-Analytic Connectivity Mapping (MACM) to relate the ALE findings to an underlying network. However, the results of the literature review on the Sleuth database meant the inability to perform such an additional analysis. Two strategies were developed: (1) to perform MACM to look at the results of current ALE meta-analysis, (2) to perform MACM for the key brain areas included in Krueger and Hoffman’s model to see the connectivity map. As suggested in GingerALE guideline and published in several papers of the developers of this software^44,45^, the search for the corresponding activations was performed via Sleuth database with the following criteria: “Context: Normal Mapping”, “Activations Only”, “Behavioral Domain: Emotion - Negative: Punishment/Loss OR Cognition: Social cognition” and for TD label we put the name of brain area of interest (“claustrum”, “inferior frontal gyrus”, “superior frontal gyrus”). After that all papers were visually inspect to see, if the described study was performed about social punishment. Unfortunately, the database revealed too few results for all keywords: only two experiments from the two articles belong to social punishment, but both of them appeared in “сlaustrum” and “inferior frontal gyrus” Sleuth searches.

Following the same steps of extracting the information from the Sleuth database for the second MACM search, only two eligible experiments were found for “insula”, no experiments for “anterior cingulate”, one experiment for “posterior cingulate”, three eligible experiments for “amygdala” and so forth. Despite such an analysis being very important improvement to this study allowing a detailed evaluation of the concordant activations and co-activations across social punishment studies, the Sleuth database does not contain enough information to perform such a study. This could be considered as a shortcoming of the current study, which should be addressed in future studies.

To sum up, the concordance across studies of social punishment has been observed in the bilateral claustrum, right interior frontal and left superior frontal gyri - areas related to salience and the central-executive network. The data obtained here on typical adults provide a stereotaxic set of brain regions which could serve as regions of interest to guide future research in this field.

## Methods

### Literature search

In comparison to previous meta-analyses conducted on fMRI results of brain activations to specific economic tasks (Ultimatum Game^46,47^; Trust Game^48^), the scope of this meta-analysis is an investigation of the general brain responses to social punishment. In accordance with Muller and colleagues^49^, to answer this research question the analysis was not limited to a specific paradigm (e.g., Ultimatum Game or Dictator Game with punishment condition), but all paradigms were considered, which allow focusing on the higher order processes necessary in all tasks related to social punishment. Importantly, it required the balancing of the distribution of experiments across tasks^49^ and consideration of the problem of the results’ generalization, because the specific types of experiments could drive the results. Therefore, the literature search was defined to account for the data reported for the behavioral phenomenon (social punishment, altruistic punishment, costly punishment) rather than specific task, such as Ultimatum Game or third-party punishment game (for the similar approach in social neuroscience meta-analyses see the following^50,51^).

The literature search was performed on the 25^th^ of May 2018 using the Web of Science Core Collection search engine (http://apps.webofknowledge.com/) and PubMed database (https://www.ncbi.nlm.nih.gov/pubmed/) using the following keywords: “fMRI AND social punishment”, “fMRI AND costly punishment”, and “fMRI AND altruistic punishment”. The search yielded 211 articles. The search was updated on 15^th^ of April, 2019 to include articles published and included into databases from 25^th^ of May, yielding 222 articles. After removing duplicates and non-full-text articles, the articles were screened for eligibility using the following exclusion criteria: articles reporting data on children, patients and drug administration, no fMRI data or only region of interest (ROI) data, between-group analysis, non-relevant tasks or review articles were excluded (see Fig. 1 for the PRISMA chart). Only studies reporting whole-brain analysis results in Talairach or Montreal Neurological Institute (MNI) spaces for healthy, human adults were included. Due to the different spatial and temporal resolutions between fMRI, positron emission tomography (PET) and magnetoencephalography (MEG), only data from fMRI studies were included, while PET and MEG studies were excluded from the search criteria to achieve the homogeneity of imaging data. The articles eligible for the meta-analysis were also screened for the tasks eligible to avoid possible overlapping with the results of previous study on brain responses to social norms and its violations^11^ and to confirm the same contrasts would not be included into different analyses. This yielded 17 articles with 18 contrasts, and 191 foci eligible for meta-analysis.

### Article selection

The main objective of this meta-analysis is to understand how the brain processes information regarding altruistic or social punishment. To achieve this, studies that reported brain activations as a response to negative outcomes (i.e. game losses vs. wins only) were not included; only those that reported them in the context of social situations were included, such those using fairness-related norm tasks (Ultimatum Game, Dictator Game) and social rejection tasks (Cyberball game, modified social incentive delay task). While for second-party punishment the main form of punishment decision was rejection behaviour (directly leading to financial losses of the wrongdoer or perceived as a social disapproval - an unpleasant outcome), the third-party punishment decisions included different forms of punishment behaviour: from direct investment of the third-party’s own resources in economic tasks to punishment ratings evaluating the power of punishment assigned to the wrongdoer in criminal scenarios.

The majority of studies used fairness-related norm tasks and neuroeconomic paradigms, such as Ultimatum Game or third-party punishment game (61%), while three used norm-violation scenarios and vignettes with a punishment questionnaire^52–54^, one used social incentive delay tasks^55^, one used the Cyberball paradigm with punishment questionnaire^56^ and one a perceptual task with social feedback^57^. The insufficient number of experiments (<17) did not allow for examining concordance in the subcategories for second-party punishment and third-party punishment^43^. In the following studies, participants were made to experience punishment in the form of social disapproval^55,57^ or direct financial losses^58^, while another afforded participants the possibility to punish the inappropriate behaviour of another person (for instance, assign the punishment rating or reject the proposed offer)^7,52–54,56,59–67^.

### ALE meta-analysis

This coordinate-based meta-analysis was conducted using GingerALE software (2.3.6), freely available on http://www.brainmap.org/ale/. This method allows using foci from different articles to create a probabilistic map of activations that is compared to random spatial distributions. The significance of the data has been assessed using a cluster-level threshold for multiple comparisons at p = 0.05 with a cluster-forming threshold set to p = 0.001^43,68^.

### Ethical approval

This article does not contain any studies with human participants directly performed by any of the authors.

## Supplementary information

PRISMA checklist

## Data Availability

The datasets analysed during the current study are available from the corresponding author on reasonable request.

## References

[1] Fehr E, Fischbacher U, Gächter S (2002). Strong reciprocity, human cooperation, and the enforcement of social norms. Hum Nat..

[2] Li X (2017). Punishment diminishes the benefits of network reciprocity in social dilemma experiments. Proc Natl Acad Sci USA.

[3] Fehr E, Gächter S (2002). Altruistic punishment in humans. Nature.

[4] Fehr E, Fischbacher U (2004). Third-party punishment and social norms. Evol. Hum. Behav..

[5] Hoffman, M. *The Punisher’s Brain*. (Cambridge University Press, 2014).

[6] Krueger F, Hoffman M (2016). The Emerging Neuroscience of Third-Party Punishment. Trends in Neurosciences.

[7] Strobel A (2011). Beyond revenge: neural and genetic bases of altruistic punishment. Neuroimage.

[8] Stallen M (2018). Neurobiological Mechanisms of Responding to Injustice. J. Neurosci.

[9] Civai C, Miniussi C, Rumiati RI (2015). Medial prefrontal cortex reacts to unfairness if this damages the self: a tDCS study. Social Cognitive and Affective Neuroscience.

[10] Civai C, Huijsmans I, Sanfey AG (2019). Neurocognitive mechanisms of reactions to second- and third-party justice violations. Scientific Reports.

[11] Zinchenko O, Arsalidou M (2018). Brain responses to social norms: meta-analyses of fMRI studies. Hum. Brain Mapp..

[12] Smith VL (1982). Microeconomic systems as an experimental science. American Economic Review.

[13] Guala F (2012). Reciprocity: weak or strong? What punishment experiments do (and do not) demonstrate. Behav Brain Sci..

[14] Engen A (2014). Communication, Expression, and the Justification of Punishment. Athens Journal of Humanities & Arts.

[15] Ginther MR (2016). Parsing the Behavioral and Brain Mechanisms of Third-Party Punishment. The Journal of neuroscience: the official journal of the Society for Neuroscience.

[16] Buckholtz JW, Marois R (2012). The roots of modern justice: cognitive and neural foundations of social norms and their enforcement. Nature Neuroscience.

[17] Carter CS (1998). Anterior cingulate cortex, error detection, and the online monitoring of performance. Science.

[18] Botvinick MM, Cohen JD, Carter CS (2004). Conflict monitoring and anterior cingulate cortex: an update. Trends Cogn Sci..

[19] Swick D, Turken AU (2002). Dissociation between conflict detection and error monitoring in the human anterior cingulate cortex. Proc Natl Acad Sci USA.

[20] Jabbi M, Keysers C (2008). Inferior frontal gyrus activity triggers anterior insula response to emotional facial expressions. Emotion.

[21] Perry D, Hendler T, Shamay-Tsoory SG (2012). Can we share the joy of others? Empathic neural responses to distress vs joy. Social Cognitive and Affective Neuroscience.

[22] Peled-Avron L, Glasner L, Gvirts HZ, Shamay-Tsoory SG (2019). The role of the inferior frontal gyrus in vicarious social touch: A transcranial direct current stimulation (tDCS) study. Dev Cogn Neurosci..

[23] Iacoboni M (2009). Imitation, empathy, and mirror neurons. Annu Rev Psychol..

[24] Liu T, Saito H, Oi M (2015). Role of the right inferior frontal gyrus in turn-based cooperation and competition: A near-infrared spectroscopy study. Brain and Cognition.

[25] Iacoboni M (2005). Grasping the intentions of others with one’s own mirror neuron system. PLoS Biology.

[26] Rojas DC (2006). Regional gray matter volumetric changes in autism associated with social and repetitive behavior symptoms. BMC Psychiatry.

[27] Greene DJ (2011). Atypical Neural Networks for Social Orienting in Autism Spectrum Disorders. Neuroimage.

[28] Smith JB, Radhakrishnan H, Alloway KD (2012). Rat claustrum coordinates but does not integrate somatosensory and motor cortical information. J. Neurosci..

[29] Smythies J, Edelstein L, Ramachandran V (2012). Hypotheses relating to the function of the claustrum. Front. Integr. Neurosci..

[30] Carr L, Iacoboni M, Dubeau MC, Mazziotta JC, Lenzi GL (2003). Neural mechanisms of empathy in humans: A relay from neural systems for imitation to limbic areas. Proc Natl Acad Sci USA.

[31] White MG (2018). Anterior Cingulate Cortex Input to the Claustrum Is Required for Top-Down Action Control. Cell Reports.

[32] Remedios R, Logothetis NK, Kayser C (2010). Unimodal responses prevail within the multisensory claustrum. J. Neurosci..

[33] Remedios R, Logothetis NK, Kayser C (2014). A role of the claustrum in auditory scene analysis by reflecting sensory change. Frontiers in Systems Neuroscience.

[34] Hanakawa T (2002). The role of rostral Brodmann area 6 in mental-operation tasks: an integrative neuroimaging approach. Cereb Cortex.

[35] Strotzer M (2009). One century of brain mapping using Brodmann areas. Klin Neuroradiol..

[36] Grasby PM (1994). A graded task approach to the functional mapping of brain areas implicated in auditory-verbal memory. Brain.

[37] Fletcher PC, Henson RNA (2001). Frontal lobes and human memory: insights from functional imaging. Brain.

[38] du Boisgueheneuc F (2006). Functions of the left superior frontal gyrus in humans: a lesion study. Brain.

[39] Vogel T (2016). Increased superior frontal gyrus activation during working memory processing in psychosis: Significant relation to cumulative antipsychotic medication and to negative symptoms. Schizophr Res..

[40] Cutini S (2008). Selective activation of the superior frontal gyrus in task-switching: an event-related fNIRS study. Neuroimage.

[41] Dehaene S (1996). Cerebral activations during number multiplication and comparison: a PET study. Neuropsychologia.

[42] Mellet E (1996). Functional anatomy of spatial imagery generated from verbal instructions. J Neurosci.

[43] Eickhoff SB, Laird AR, Fox PM, Lancaster JL, Fox PT (2017). Implementation errors in the GingerALE Software: description and recommendations. Hum. Brain Mapp..

[44] Robinson JL, Laird AR, Glahn DC, Lovallo WR, Fox PT (2010). Metaanalytic connectivity modeling: delineating the functional connectivity of the human amygdala. Hum Brain Mapp..

[45] Eickhoff SB (2011). Co-activation patterns distinguish cortical modules, their connectivity and functional differentiation. Neuroimage.

[46] Gabay AS, Radua J, Kempton MJ, Mehta MA (2014). The ultimatum game and the brain: A meta‐analysis of neuroimaging studies. Neuroscience & Biobehavioral Reviews.

[47] Feng C, Luo Y, Krueger F (2015). Neural signatures of fairness‐related normative decision making in the ultimatum game: A coordinate‐based meta‐analysis. Human Brain Mapping.

[48] Bellucci G, Chernyak SV, Goodyear K, Eickhoff SB, Krueger F (2017). Neural signatures of trust in reciprocity: A coordinate-based meta-analysis. Human Brain Mapping.

[49] Müller VI (2018). Ten simple rules for neuroimaging meta-analysis. Neuroscience and biobehavioral reviews.

[50] Shkurko AV (2013). Is social categorization based on relational ingroup/outgroup opposition? A meta-analysis. Social Cognitive and Affective Neuroscience.

[51] Vijayakumar N, Cheng TW, Pfeifer JH (2017). Neural correlates of social exclusion across ages: A coordinate-based meta-analysis of functional MRI studies. NeuroImage.

[52] Buckholtz JW (2008). The neural correlates of third-party punishment. Neuron.

[53] Bellucci G (2017). Effective connectivity of brain regions underlying third-party punishment: Functional MRI and Granger causality evidence. Soc Neurosci..

[54] Treadway MT (2014). Corticolimbic gating of emotion‐driven punishment. Nature Neuroscience.

[55] Kohls G (2013). The nucleus accumbens is involved in both the pursuit of social reward and the avoidance of social punishment. Neuropsychologia.

[56] Moor BG (2012). Social exclusion and punishment of excluders: neural correlates and developmental trajectories. Neuroimage.

[57] Vrtička P, Andersson F, Grandjean D, Sander D, Vuilleumier P (2008). Individual Attachment Style Modulates Human Amygdala and Striatum Activation during Social Appraisal. PLoS ONE.

[58] Spitzer M, Fischbacher U, Herrnberger B, Grön G, Fehr E (2007). The neural signature of social norm compliance. Neuron.

[59] Baumgartner T, Götte L, Gügler R, Fehr E (2012). The mentalizing network orchestrates the impact of parochial altruism on social norm enforcement. Hum Brain Mapp.

[60] Corradi-Dell’Acqua C, Civai C, Rumiati RI, Fink GR (2013). Disentangling self- and fairness-related neural mechanisms involved in the ultimatum game: an fMRI study. Social Cognitive and Affective Neuroscience.

[61] Feng C (2016). Diffusion of responsibility attenuates altruistic punishment: A functional magnetic resonance imaging effective connectivity study. Hum Brain Mapp..

[62] Guo X (2013). Increased neural responses to unfairness in a loss context. Neuroimage.

[63] Hu Y, Strang S, Weber B (2015). Helping or punishing strangers: neural correlates of altruistic decisions as third-party and of its relation to empathic concern. Frontiers in Behavioral Neuroscience.

[64] Wang L (2017). Neural substrates of context- and person-dependent altruistic punishment. Hum Brain Mapp..

[65] Wei C (2018). Social Support Modulates Neural Responses to Unfairness in the Ultimatum Game. Frontiers in Psychology.

[66] Will G-J, Crone EA, Güroğlu B (2015). Acting on social exclusion: neural correlates of punishment and forgiveness of excluders. Social Cognitive and Affective Neuroscience.

[67] Wu Y, Zang Y, Yuan B, Tian X (2015). Neural correlates of decision making after unfair treatment. Frontiers in Human Neuroscience.

[68] Eickhoff SB, Bzdok D, Laird AR, Kurth F, Fox PT (2012). Activation likelihood estimation revisited. Neuroimage.

[69] Moher D, Liberati A, Tetzlaff J, Altman DG, PRISMA Group (2009). Preferred reporting items for systematic reviews and meta-analyses: the PRISMA statement. PLoS medicine.

