# Supplementary material for: Brain responses to social punishment: a meta-analysis
Source: Sci Rep. 2019 Sep 5;9:12800. doi: 10.1038/s41598-019-49239-1 (PMC6728376; doi:10.1038/s41598-019-49239-1)
Supplement: Supplementary file 1 — PRISMA checklist [file 41598_2019_49239_MOESM1_ESM.pdf]

Brain responses to social punishment: a meta-analysis

Names and affiliations:

Oksana Zinchenko [1*]

[1] Centre for Cognition and Decision Making, Institute for Cognitive Neuroscience, National Research University Higher School of Economics, Moscow, Russian Federation

* Contact information for the corresponding author.

Address: 101000, Krivokolenniy sidewalk, 3a, Moscow

E-mail: ozinchenko@hse.ru

Work telephone number: 8 (495) 916-8900 * 22-370

ORCID: 0000-0002-7976-3224

PRISMA 2009 Checklist [69]

Section/topic	#	Checklist item	Reported on page #
**TITLE**			
Title	1	Brain responses to social punishment: a coordinate-based ALE meta-analysis	1
**ABSTRACT**			
Structured summary	2	By using coordinate-based fMRI meta-analysis, the present study examined the concordant map of neural activations associated with various social punishment tasks. A total of 17 articles with 18 contrasts including 383 participants, equalling 191 foci were included in activation likelihood estimation (ALE) analysis. The majority of the studies (61%) included employed the widely used neuroeconomic paradigms, such as fairness-related norm tasks (Ultimatum Game, third-party punishment game), while the remaining tasks reported included criminal scenarios evaluation and social rejection tasks. The analysis presented revealed concordant activation in the bilateral claustrum, right interior frontal and left superior frontal gyri.	2
**INTRODUCTION**			
Rationale	3	In addition, a recent review by Krueger and Hoffman6 provides us with a neural framework for punishment that could offer some insights regarding the involvement of large-scale networks supporting third-party punishment: the salience network anchored in the anterior cingulate cortex, the mentalizing network – in the temporoparietal junction and dorsomedial prefrontal cortex and central-executive network – in the dorsolateral prefrontal cortex. Recent fMRI study by Civai and colleagues10 suggests that exploration of the similarities in neural processing of second-party and third-party punishment conditions could deep our knowledge in disentangling mechanisms associated with general punishment processes. However, no extensive review or meta-analytic study has been done on social punishment to address shared brain mechanisms for both second- and third-party punishment: a recent ALE meta-analysis was conducted on social norm representation and norm violations11, revealing the distinct brain regions responsible for these two processes – the anterior cingulate cortex, medial frontal gyrus and insular cortex. The majority of the experiments testing social punishment focus on punishment in terms of direct material costs alone. However, initially12 this has been done to control individual incentives and test exact theoretical predictions, while laboratory behaviour of meeting and avoiding punishment is similar to the disapproval and its avoidance in real life. Thus, social punishment should be seen more as different forms of social control 13, or judgments of disapproval and negative emotional responses to the norm violator14, so the meta-analytic research should follow this logic to better estimate concordant activation to social punishment. Following this, the search for the current study was constructed to address different terms used to describe social punishment: social punishment, altruistic punishment, costly punishment - so different tasks from the punishment experiments would be included in the study to replicate a mechanism that supports cooperation and enforces social norms. The initial literature search performed in April, 2017, did not reveal enough studies eligible to be included in the analysis of general brain responses to social	3-4

		punishment.	
Objectives	4	This study is an attempt to address this lack of knowledge and identify the concordant brain activations responsible for the processing of information related to social punishment.	4
**METHODS**			
Protocol and registration	5	-	-
Eligibility criteria	6	The articles were screened for eligibility using the following exclusion criteria: articles reporting data on children, patients and drug administration, no fMRI data or only region of interest (ROI) data, between-group analysis, non-relevant tasks or review articles were excluded (see Figure 1 for the PRISMA chart). Only studies reporting whole-brain analysis results in Talairach or Montreal Neurological Institute (MNI) spaces for healthy, human adults were included. Due to the different spatial and temporal resolutions between fMRI, positron emission tomography (PET) and magnetoencephalography (MEG), only data from fMRI studies were included	8
Information sources	7	The literature search was performed on the 25th of May 2018 using the Web of Science Core Collection search engine (http://apps.webofknowledge.com/) and PubMed database (https://www.ncbi.nlm.nih.gov/pubmed/) using the following keywords: “fMRI AND social punishment”, “fMRI AND costly punishment”, and “fMRI AND altruistic punishment”. The search yielded 211 articles. The search was updated on 15th of April, 2019 to include articles published and included into databases from 25th of May, yielding 222 articles.	8-9
Search	8	The literature search was performed on the 25th of May 2018 using the Web of Science Core Collection search engine (http://apps.webofknowledge.com/) and PubMed database (https://www.ncbi.nlm.nih.gov/pubmed/) using the following keywords: “fMRI AND social punishment”, “fMRI AND costly punishment”, and “fMRI AND altruistic punishment”. The search yielded 211 articles. The search was updated on 15th of April, 2019 to include articles published and included into databases from 25th of May, yielding 222 articles. After removing duplicates and non-full-text articles, the articles were screened for eligibility using the following exclusion criteria: articles reporting data on children, patients and drug administration, no fMRI data or only region of interest (ROI) data, between-group analysis, non-relevant tasks or review articles were excluded (see Figure 1 for the PRISMA chart). Only studies reporting whole-brain analysis results in Talairach or Montreal Neurological Institute (MNI) spaces for healthy, human adults were included. Due to the different spatial and temporal resolutions between fMRI, positron emission tomography (PET) and magnetoencephalography (MEG), only data from fMRI studies were included, while PET and MEG studies were excluded from the search criteria to achieve the homogeneity of imaging data. The articles eligible for the meta-analysis were also screened for the tasks eligible to avoid possible overlapping with the results of previous study on brain responses to social norms and its violations11 and to confirm the same contrasts would not be included into different analyses. This yielded 17 articles with 18 contrasts, and 191 foci eligible for meta-analysis.	8-9
Study selection	9	The main objective of this meta-analysis is to understand how the brain processes information regarding altruistic or social punishment. To achieve this, studies that reported brain activations as a response to negative outcomes (i.e.	9

		game losses vs. wins only) were not included; only those that reported them in the context of social situations were included, such those using fairness-related norm tasks (Ultimatum Game, Dictator Game) and social rejection tasks (Cyberball game, modified social incentive delay task). While for second-party punishment the main form of punishment decision was rejection behaviour (directly leading to financial losses of the wrongdoer or perceived as a social disapproval - an unpleasant outcome), the third-party punishment decisions included different forms of punishment behaviour: from direct investment of the third-party's own resources in economic tasks to punishment ratings evaluating the power of punishment assigned to the wrongdoer in criminal scenarios.	
Data collection process	10	The literature search was performed on the 25th of May 2018 using the Web of Science Core Collection search engine (http://apps.webofknowledge.com/) and PubMed database (https://www.ncbi.nlm.nih.gov/pubmed/) using the following keywords: "fMRI AND social punishment", "fMRI AND costly punishment", and "fMRI AND altruistic punishment". The search yielded 211 articles. The search was updated on 15th of April, 2019 to include articles published and included into databases from 25th of May, yielding 222 articles.	8
Data items	11	The articles eligible for the meta-analysis were also screened for the tasks eligible to avoid possible overlapping with the results of previous study on brain responses to social norms and its violations11 and to confirm the same contrasts would not be included into different analyses.	8
Risk of bias in individual studies	12	The data presented here represent concordant neural activation across studies that mostly investigated the brain responses of punishers (fifteen studies out of seventeen) using neuroeconomic paradigms (eleven out of seventeen). The optimal approach would be possible with the further implementation of subcategories (i.e. second- vs third-party punishment; experience of receiving the punishment vs application of the punishment to the other person). In accordance with current guidelines, the number of experiments in these subcategories does not allow us to continue the examination of the concordance.	7
Summary measures	13	Articles included in the meta-analysis provided data on 383 participants (see Table 1). Five articles did not report the gender of their final sample after all exclusions; of the remaining articles, 57% were female participants. Four articles did not report handedness (25%); of the remaining articles all tested participants were right-handed (100%). Five articles did not report the age of the final sample of the participants; of the remaining articles, the mean age range was 19.76-28.39 years. 50% of the articles did not report the education level of participants, while 100% of the participants of the remaining articles were reported to have some university education. Figure 1 shows the number of articles, number of studies (experiments) and number of foci included in the meta-analysis.	4
Synthesis of results	14	Two clusters were detected during the analysis (see Figure 2, Table 2; the coordinates are in Talairach space). One with the highest likelihood of detection includes right claustrum and right inferior frontal gyrus (BA 45). Other regions include the left claustrum and left superior frontal gyrus (BA 6).	5

Section/topic	#	Checklist item	Reported on page #

Risk of bias across studies	15	The data presented here represent concordant neural activation across studies that mostly investigated the brain responses of punishers (fifteen studies out of seventeen) using neuroeconomic paradigms (eleven out of seventeen). The optimal approach would be possible with the further implementation of subcategories (i.e. second- vs third-party punishment; experience of receiving the punishment vs application of the punishment to the other person). In accordance with current guidelines, the number of experiments in these subcategories does not allow us to continue the examination of the concordance.	9
Additional analyses	16	-	-
**RESULTS**			
Study selection	17	The search yielded 211 articles. The search was updated on 15th of April, 2019 to include articles published and included into databases from 25th of May, yielding 222 articles. After removing duplicates and non-full-text articles, the articles were screened for eligibility using the following exclusion criteria: articles reporting data on children, patients and drug administration, no fMRI data or only region of interest (ROI) data, between-group analysis, non-relevant tasks or review articles were excluded (see Figure 1 for the PRISMA chart). Only studies reporting whole-brain analysis results in Talairach or Montreal Neurological Institute (MNI) spaces for healthy, human adults were included. Due to the different spatial and temporal resolutions between fMRI, positron emission tomography (PET) and magnetoencephalography (MEG), only data from fMRI studies were included, while PET and MEG studies were excluded from the search criteria to achieve the homogeneity of imaging data. The articles eligible for the meta-analysis were also screened for the tasks eligible to avoid possible overlapping with the results of previous study on brain responses to social norms and its violations10 and to confirm the same contrasts would not be included into different analyses. This yielded 17 articles with 18 contrasts, and 191 foci eligible for meta-analysis.	9, Figure 1 (flow diagram)
Study characteristics	18	The majority of studies used fairness-related norm tasks and neuroeconomic paradigms, such as Ultimatum Game or third-party punishment game (61%), while three used norm-violation scenarios and vignettes with a punishment questionnaire53 54 55, one used social incentive delay tasks56, one used the Cyberball paradigm with punishment questionnaire57 and one a perceptual task with social feedback58. The insufficient number of experiments (<17) did not allow for examining concordance in the subcategories for second-party punishment and third-party punishment43. In the following studies, participants were made to experience punishment in the form of social disapproval56 58 or direct financial losses59, while another afforded participants the possibility to punish the inappropriate behaviour of another person (for instance, assign the punishment rating or reject the proposed offer).8 53 54 55 57 60 61 62 63 64 65 66 67 68	9, Table 1
Risk of bias within studies	19	-	-
Results of individual studies	20	-	-

Synthesis of results	21	Two clusters were detected during the analysis (see Figure 2, Table 2; the coordinates are in Talairach space). One with the highest likelihood of detection includes right claustrum and right inferior frontal gyrus (BA 45). Other regions include the left claustrum and left superior frontal gyrus (BA 6).	5
Risk of bias across studies	22	The data presented here represent concordant neural activation across studies that mostly investigated the brain responses of punishers (fifteen studies out of seventeen) using neuroeconomic paradigms (eleven out of seventeen). The optimal approach would be possible with the further implementation of subcategories (i.e. second- vs third-party punishment; experience of receiving the punishment vs application of the punishment to the other person). In accordance with current guidelines, the number of experiments in these subcategories does not allow us to continue the examination of the concordance.	9
Additional analysis	23	-	-
**DISCUSSION**			
Summary of evidence	24	The analysis revealed concordant bilateral activation in the claustrum, left superior frontal gyrus and right inferior frontal gyrus for social punishment tasks. The former corresponds to the region next to the insular cortex, which is considered in Krueger and Hoffman's model as part of the salience network. The latter corresponds to the dorsolateral and ventrolateral prefrontal cortex, respectively, while the dorsolateral prefrontal cortex represents the main node of central-executive network in Krueger and Hoffman's model. It needs to be taken into account that the analysis did not reveal concordant activation in any regions corresponding with the mentalizing network from the model. TPJ is a key node of the mentalizing network included in the model, whose activity is associated with the evaluation of the wrongdoer's mental state and his/her intention 6 15. This could be explained by the heterogeneity of the studies included: the proportion of third-party and second-party punishment tasks is not equal, and it has been shown that second-party and third-party punishment tasks trigger different responses in the mentalizing system 9 16. The analysis did not reveal any concordant activation in ACC responsible for the initial evaluation of harm to the victim of the norm violation, according to the Krueger and Hoffman model. In general ACC activity is associated with error detection and performance monitoring 17 18 19. For social punishment, this suggests that ACC activity is required for the initial stages of punishment decision-making, like the evaluation of the current norm and a detection of the norm violation, which is consistent also with the results of the recent meta-analysis11. As most studies included in the current meta-analysis used the contrasts where the participants were faced with the punishment decision of others / make the punishment decision by themselves (assigning the rating, deducting the monetary units etc.), this could suggest that the final stages of punishment decision-making only require already available information about the harm done to the victim (associated with the insular/claustrum activity) to make the final decision (which involves lateral prefrontal cortex/inferior frontal gyrus activity).<br>The right inferior frontal gyrus is considered as a key region in the emotional empathy network20 21 22 23. The activation of this region has been also associated with interpretation during interpersonal interactions24 and an understanding of the intentions of others25. The structural abnormalities of this region have been reported in association with the severity of symptoms in autism spectrum disorders, such as reduced cortical thickness26 or the	5-7

		absence of functional activation27 . Based on these findings, it could be suggested that the activation of this part of the ventrolateral prefrontal cortex reflects the activation of the emotional empathy network required for the adequate response to social interaction.<br>According to a recent meta-analysis, the concordant activation of the left claustrum is associated with norm violation tasks11, although its direct role in social cognition is still unclear. However past hypotheses have suggested the role of the claustrum in mammals as an integrator of multimodal sensory information, though some recent findings do not confirm this notion28 29; this suggests its involvement in top-down control and encoding a preparatory signal in rat models28.<br>It is important to note that emotional empathy networks, based on the concept of mirror neuron systems, are now claimed to include regions of the somatosensory cortex and anterior insula30. Recent findings suggest that the right inferior frontal gyrus response triggers the activity in the anterior insula associated with emotional simulation20, confirming its interaction during the evaluation of social-emotional events. Moreover, some researchers claim that the role of the anterior insula as a hub of salience networks could be confounded by its proximity to the claustrum31, which is supported by findings on monkeys32 33, suggesting that claustrum reacts to the novel and salient stimuli. Based on this, it could be suggested that activations of the claustrum in social punishment tasks are a function of salience detection.<br>The analysis also revealed the concordant activation in left superior frontal gyrus (BA 6) for social punishment. This area could be divided into three functional subdivisions - supplementary motor area, the dorsal and ventral parts of lateral premotor cortex34. The findings suggest that various tasks could activate different parts of this area. The medial part of BA 6 corresponds to the supplementary motor area, whose main functions are motor learning and planning35, however, as part of DLPFC, is it also involved in working memory36 37. Lesion studies suggest that the impairment of the left superior frontal gyrus is associated with working memory deficits38. Schizophrenia patient studies also confirm the role of this region in working memory, showing a higher activation of the left superior frontal gyrus during more complex working memory tasks39. The involvement of the activity of this region in working memory tasks is also supported by findings on healthy subjects, addressed in particular to task-switching40. The rostral part of BA 6, however, is activated in mental-operation tasks without motor responses involved34. Some studies also linked BA 6's activation with number multiplication and comparison41 and spatial imagery42, which suggests that the activation of this area could be responsible for the interaction of cognitive information processing and motor control.	
Limitations	25	The current study employed ALE approach to investigate concordant activations to social punishment, following the model of Krueger and Hoffman. An alternative solution would be to perform additional analysis, such as Meta-Analytic Connectivity Mapping (MACM) to relate the ALE findings to an underlying network. However, the results of the literature review on the Sleuth database meant the inability to perform such an additional analysis. Two strategies were developed: 1) to perform MACM to look at the results of current ALE meta-analysis, 2) to perform MACM for the key brain areas included in Krueger and Hoffman's model to see the connectivity map. As suggested in GingerALE guideline and published in several papers of the developers of this software44 45, the search for the corresponding activations was performed via Sleuth database with the following criteria: "Context: Normal Mapping", "Activations	7-8

		Only", "Behavioral Domain: Emotion - Negative: Punishment / Loss OR Cognition: Social cognition" and for TD label we put the name of brain area of interest ("claustrum", "inferior frontal gyrus", "superior frontal gyrus"). After that all papers were visually inspect to see, if the described study was performed about social punishment. Unfortunately, the database revealed too few results for all keywords: only two experiments from the two articles belong to social punishment, but both of them appeared in "claustrum" and "inferior frontal gyrus" Sleuth searches.<br>Following the same steps of extracting the information from the Sleuth database for the second MACM search, only two eligible experiments were found for "insula", no experiments for "anterior cingulate", one experiment for "posterior cingulate", three eligible experiments for "amygdala" and so forth. Despite such an analysis being very important improvement to this study allowing a detailed evaluation of the concordant activations and co-activations across social punishment studies, the Sleuth database does not contain enough information to perform such a study. This could be considered as a shortcoming of the current study, which should be addressed in future studies.	
Conclusions	26	To sum up, the concordance across studies of social punishment has been observed in the bilateral claustrum, right interior frontal and left superior frontal gyri - areas related to salience and the central-executive network. The data obtained here on typical adults provide a stereotaxic set of brain regions which could serve as regions of interest to guide future research in this field.	8
**FUNDING**			
Funding	27	The article was prepared within the framework of the HSE University Basic Research Program and funded by the Russian Academic Excellence Project '5-100'.	18

PRISMA 2009 Checklist [69]

From: Moher D, Liberati A, Tetzlaff J, Altman DG, The PRISMA Group (2009). Preferred Reporting Items for Systematic Reviews and Meta-Analyses: The PRISMA Statement. PLoS Med 6(7): e1000097. doi:10.1371/journal.pmed1000097

For more information, visit: **www.prisma-statement.org**.
